# Could In-Home Sensors Surpass Human Observation of People with Parkinson's at High Risk of Falling? An Ethnographic Study

**DOI:** 10.1155/2016/3703745

**Published:** 2016-02-14

**Authors:** Emma Stack, Rachel King, Balazs Janko, Malcolm Burnett, Nicola Hammersley, Veena Agarwal, Sion Hannuna, Alison Burrows, Ann Ashburn

**Affiliations:** ^1^Faculty of Health Sciences, University of Southampton, Southampton SO17 1BJ, UK; ^2^NIHR CLAHRC Wessex, University of Southampton, Southampton SO17 1BJ, UK; ^3^School of Systems Engineering, University of Reading, Reading RG1 5AQ, UK; ^4^Faculty of Medicine, University of Southampton, Southampton SO17 1BJ, UK; ^5^Faculty of Engineering, University of Bristol, Bristol BS8 1TH, UK

## Abstract

Self-report underpins our understanding of falls among people with Parkinson's (PwP) as they largely happen unwitnessed at home. In this qualitative study, we used an ethnographic approach to investigate* which* in-home sensors, in* which* locations, could gather useful data about fall risk. Over six weeks, we observed five independently mobile PwP at high risk of falling, at home. We made field notes about falls (prior events and concerns) and recorded movement with video, Kinect, and wearable sensors. The three women and two men (aged 71 to 79 years) having moderate or severe Parkinson's were dependent on others and highly sedentary. We most commonly noted balance protection, loss, and restoration during chair transfers, walks across open spaces and through gaps, turns, steps up and down, and tasks in standing (all evident walking between chair and stairs, e.g.). Our unobtrusive sensors were acceptable to participants: they could detect instability during everyday activity at home and potentially guide intervention. Monitoring the route between chair and stairs is likely to give information without invading the privacy of people at high risk of falling, with very limited mobility, who spend most of the day in their sitting rooms.

## 1. Background

People at high risk of falling spend most of their time at home, and, like many other manifestations of illness, falls happen predominantly unwitnessed at home. Therefore, our understanding of what happens* before, during, and after* a fall is largely dependent on self-report (predominantly through interviews, diaries, and surveys). As Weis et al. [1] stated, “Unfortunately, self-report is… the gold-standard for characterizing and quantifying fall frequency” but authors discuss the accuracy of patient recall as a limitation of their work across a range of conditions [2–7].

Costing over £2 billion per year, falls are an NHS priority, with 30% of people aged 65 or older falling each year, and 50% of those aged 80 or older [8]. “Unless concerted action is taken,” falls are likely to become increasingly prevalent and costly as the population ages [9], despite our current understanding of the risk factors and circumstances in which people tend to fall. We need to understand more about* near-misses* (“occasions on which individuals felt that they were going to fall but did not actually do so” [10]),* falls* (“events that results in a person coming to rest unintentionally on the ground or another lower level, not as the result of a major intrinsic event or overwhelming hazard” [11]), and the* fear of falling* to manage their causes, consequences, and costs [8, 10].

The quality and quantity of self-report depend on the interviewee's and interviewer's motivations and abilities. Transient risk factors (such as dizziness) contribute to falls, fear of falling, morbidity, and dependence [9] but fleeting signs of impending instability are difficult to describe and evaluate (and therefore manage), unlike the obvious signs of landing (injuries and environmental disruption). Someone who has fallen may not know what happened, let alone why, as someone falls, by definition “unintentionally,” while their attention is elsewhere. Even clear insights may fade without immediate reporting or documentation. Some people may not want to report every (or any) event. To summarise, the drawbacks of relying on self-reporting to understand fall-events include

- overreliance on a single witness whose attention was elsewhere during an unexpected event,
- vague/transient warning signs that gave insufficient insight at the time to prevent a fall (or soften a landing) probably leaving minimal evidence afterwards,
- the fact that if people want to document and/or report events, they need an opportunity to record or recall the details before insights diminish.

User-friendly, minimally invasive video-based or wearable sensors in the home could tackle many of these issues. They could, for example, record deviations from normal gait that a human observer might not notice, let alone document. Sensors could be “a virtual witness,” recording the circumstances that precede, surround, and follow fall-events. By recording a baseline, deviations, and fall-events, sensors could enhance the management and self-management of fall risk and inform clinicians about instability associated with fleeting symptoms that are difficult to recount. Beyond the individual/clinical application, information from sensors (that individuals “control” and are willing to share) could change our current thinking about the evolution of fall risk over time, the circumstances of falling, and behavioural change after falling.

For our understanding of falls to improve, we need to observe many real events. Some fall-detection algorithms probably perform so poorly in the field, for example, 85 false alarms per day [12], because researchers developed them from data collected on simulations. Volunteers throwing themselves to the ground (e.g., as Bourke et al. asked them to do [13]) do not land unexpectedly. It would not require many in-home sensors to capture more “real” falls than researchers have witnessed to date, generating data that could refine detection algorithms. Beyond simply capturing the mechanics of an event, sensors could help us to understand what happens beforehand. Cameras showed, for example, that more falls in care homes occurred from standing and while transferring and fewer during walking than reports suggested [14].

Understanding what happens before balance is lost has a preventive value. Understanding what happens afterwards has value in preventing the deleterious consequences of immobility and fear, such as isolation and dependence. But identifying what people at risk of falling do at home and how to extract useful data under the less-than-ideal conditions of the domestic setting are challenges. Deciding where to position the minimum number of sensors capable of capturing useful data, unobtrusively, in appropriate locations requires consideration. When Feldwieser et al. [15] trialled a fall-detection system in elderly people's homes, 15 falls occurred (over 1000-plus measurement days) but none within range of the Kinect sensor installed; algorithms falsely detected multiple falls every day (4592 in total); and the participants' acceptance of technology they considered “generally useful” before installation decreased with experience. To avoid some of these unwanted outcomes, we proposed a qualitative study to initiate our programme of research.

We planned to investigate the healthcare applications of a sensor platform in the home (predominantly with elderly people, as they make the greatest use of health services). People with Parkinson's (PwP) are a very high-risk group for falling at home; near-misses may herald the onset of significant postural instability and predict future falls [10, 16, 17]. If sensors could alert them to increasingly frequent near-misses at home, individuals with the most potential to benefit from rehabilitation [18] could instigate intervention before injurious falls became likely. We began our programme with an ethnographic study involving a small group of people with significant healthcare needs. “Home-based technologies research with older adults needs to be flexible and paced to fit their lives” [19], so we sought to gain insight into living at high risk of falling, attitudes to in-home sensors, and the practicalities of testing sensors under real-life conditions. We aimed to observe people with moderate or severe Parkinson's in their own homes to identify what types of sensors, and in which locations, were capable of monitoring mobility and balance in a way that would be acceptable to participants and meet the researchers' needs.

*Objectives*. The objectives were as follows:

1. To observe people at high risk of falling moving freely at home, noting, and recording (with video, Kinect, and wearable devices):
  - movement patterns (e.g., habitual activities),
  - behaviours (likely to increase or decrease fall risk),
  - locations and actions associated with (historic or observed) falls and near-misses (collectively “fall-events” [10]) and fear of falling.
2. To observe participants repeatedly demonstrating one habitual activity they associate with a particularly high fall risk (e.g., descending steps), recording from multiple camera positions.

## 2. Methods

With Ethics Committee approval, we distributed information packs to people with Parkinson's (via presentations to support groups), aiming to recruit the first five volunteers who could

1. walk at home without the assistance of another person,
2. describe multiple recent fall-events that caused them to fear falling (or falling again).

We visited potential participants to secure their written informed consent and the consent of anyone else likely to be video-recorded (e.g., a spouse at home while we were recording).

### 2.1. Data Collection and Analysis

**Figure 1:**
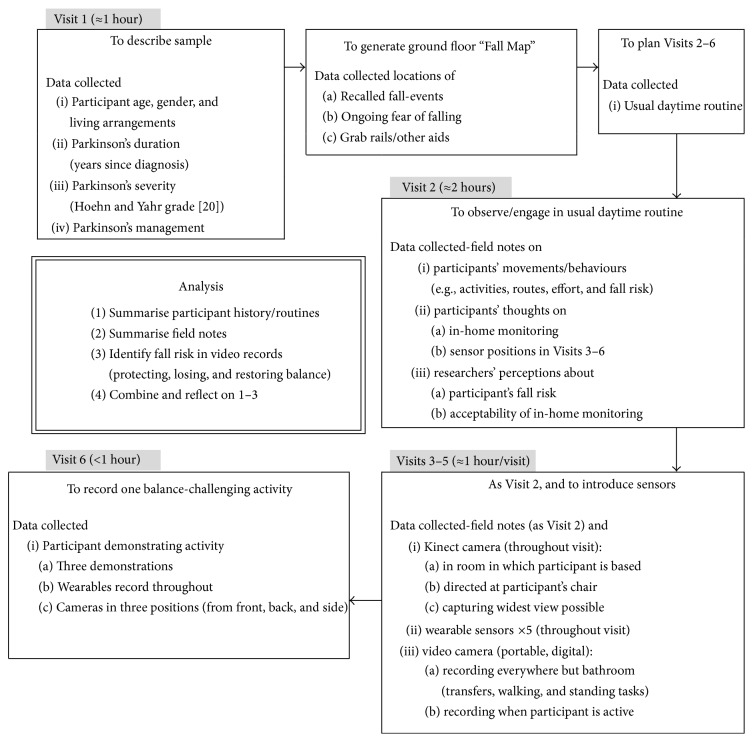
Summary of the data collection and analysis process.

Between September 2014 and February 2015, we saw participants six times (approximately weekly), engaging in their usual morning and afternoon routines at home (see Figure 1). From Visit 3, we supplemented real-time observation with video/audio recording while we were present.

**Figure 2:**
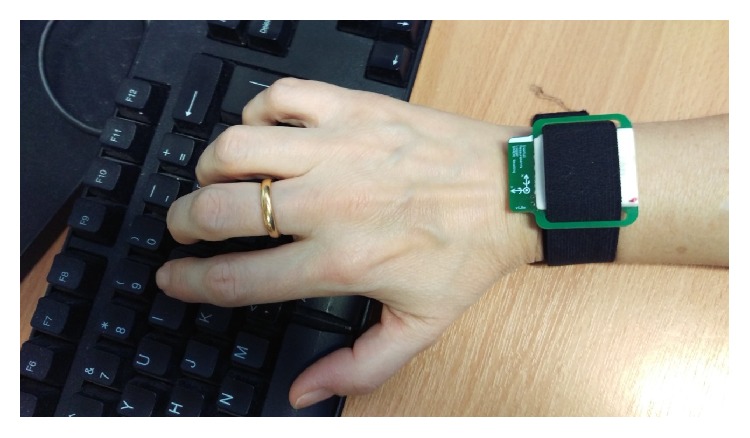
Prototype inertial measurements logger (as worn (×5) by participants).

Although we explored the use of Kinect and wearable sensors with each participant, this paper reports on only the qualitative data (derived from field notes and video review). Participants wore five self-contained watch-sized devices that were under development for a larger research collaboration (of which this study forms part) and not commercially available (see Figure 2). Each contained a triaxial accelerometer and triaxial gyroscope to measure accelerations and angular velocities. The devices ran on battery power throughout data collection and logged data that we downloaded to a computer for later analysis. We charged them fully before use and secured them around the wrists and ankles and over the lumbar spine using Velcro straps.

Visit 6 focused on an activity frequently challenging participants' balance (identified from their history and our observations). A physiotherapist annotated the videos, identifying when and how participants (1) protected, (2) lost, and (3) restored their balance (e.g., used support, swayed or stumbled, and made saving reactions).

## 3. Results

### 3.1. Sample

One participant withdrew after consenting, concerned about fitting data collection into a busy family and working life. Five completed the study, including two falling at least monthly and one with an implanted deep brain stimulator. Our participants were all retired, living alone or with a spouse, and largely housebound without assistance. We summarise their characteristics in Table 1. Three had significant healthcare needs besides Parkinson's, including neurological conditions, recurrent infections, skeletal deformities, and chronic pain and one had a spouse with significant needs. All five participants

- were under the care of Parkinson's specialists and multidisciplinary teams and followed their regular regime of “anti-Parkinson” medication throughout the study, so we observed them at peak dose (moving most freely) and as the effects wore off,
- typically spent the day in a favourite chair in their reception rooms (watching television or using a computer, e.g.) and/or sitting in the kitchen,
- described frequent near-misses and a fear of falling, at best making them “*cautious*,” at worst “*inhibitory*.”

**Figure 3:**
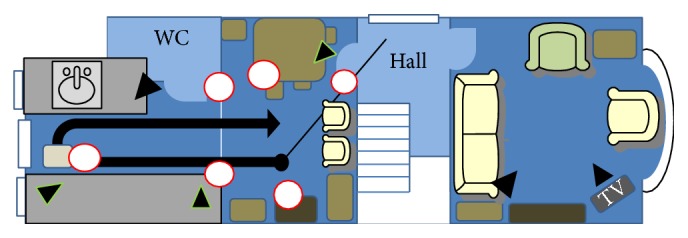
Example of Fall Map. Solid arrow shows a route through the kitchen-dining room that frequently challenges one participant; circles mark significant previous fall-events. A step between what were previously two rooms is less hazardous since the addition of grab rails on both sides. However, the participant relies on a heavy chair to provide additional support. Triangles mark camera positions.

Although in most falls at home the participants had not sustained serious injuries and had recovered to their feet without anyone's assistance, there were traumatic exceptions: one had fallen backwards from the top to the bottom of their staircase; another had fractured a femur and waited an hour, alone on the ground, for assistance. We highlight the locations associated with one participant's fall-events and fear of falling on a Fall Map in Figure 3.

### 3.2. Observation in the Home: Field Notes

#### 3.2.1. Participant's Behaviour

**Figure 4:**
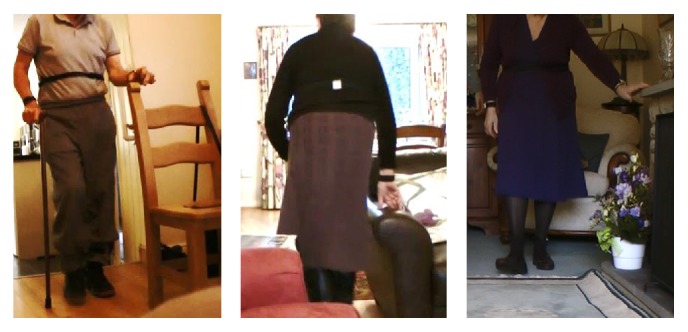
Examples of people at high risk of falling “furniture creeping.” With or without walking aids, participants relied on the support of furniture to move safely across rooms and often appeared vulnerable in open space.

We spent approximately seven hours with each participant, during which time they were all largely sedentary (staying downstairs throughout the day, mostly in one favourite chair). We positioned the Kinect in the sitting room, facing the participants' favourite chairs, except once when we focused on the computer station in the dining room. They predominantly used the furniture or walls for support (rather than mobility aids or purposely fitted rails, see Figure 4) as they showed us around their homes and gardens and demonstrated the following activities:

- Walking between rooms (e.g., to collect something or to relocate).
- Preparing drinks or cooking.
- Sorting, washing, and hanging out clothes.
- Ascending and descending stairs.
- Negotiating steps between rooms.
- Crossing open spaces in large rooms.

#### 3.2.2. Participant's Thoughts

Every participant agreed to wear five sensors throughout every session; no one said they were cumbersome; some remarked they had forgotten that they were wearing them. Participants asked about the sensors' functions and potential uses. One, who had always detested “being watched” (e.g., by a supervisor at work), felt some people might not welcome long-term video-surveillance at home. Another felt that, as carers rarely went out, sending them alerts about every fall might make them feel they had to hurry back, when that was rarely what the faller needed or wanted (as fallers keep some falls to themselves).

#### 3.2.3. Researcher's Perceptions

We felt intrusive staying “too long,” aware that people had saved tasks for when we were present, giving us “something to film.” We restricted most visits to 90 minutes, aware that participants and other residents might feel uncomfortable saying they were tired or needed privacy. Every participant was at high risk of falling (one spouse had documented approximately 30 falls over 18 months) and we* felt *anxious when they lost their balance (e.g.,* “oops, nearly!”*) or mentioned previous events (e.g., “*this is where I had my last really bad fall*”). No one fell when we were present but we observed near-misses and remained vigilant throughout. Participants frequently recounted falls with a sense of humour and told us we were being* overcautious*. Two avoided using any support despite severe instability, even when demonstrating an activity associated with previous fall-events (which appeared the only physically demanding aspect of the study, though every participant was willing to do it). Participants also seemed comfortable with how we applied sensors. The wearables did not appear to hinder or distract them but some participants appeared unstable during (or fatigued by) the repeated sitting to standing actions necessary for the application of wearables. Our greatest concern about the wearables and the Kinect was that our uncertainty about whether they were recording diverted our attention from the participants.

### 3.3. Video Analysis: Observed Risk of Falling

**Figure 5:**
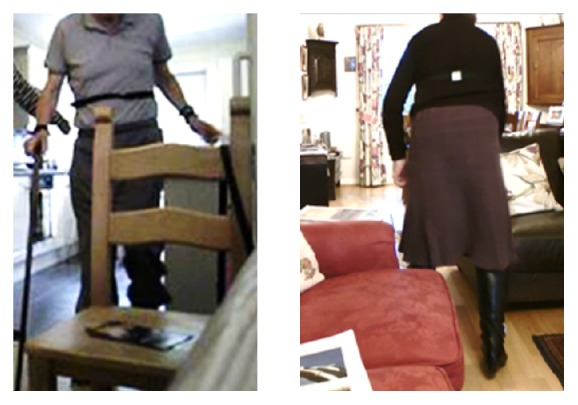
Examples of furniture obscuring the camera and challenging balance. Monitoring transfer into chairs and manoeuvring through gaps between furniture pieces would be informative as these activities frequently challenge balance. The obscured camera view highlights the importance of wearable devices as part of a sensor array.

We reviewed 246 minutes of participant activity. Although occasionally they moved out of camera view or something/someone obscured our view (see Figure 5), we counted 227 occasions when a participant appeared at imminent risk of falling (see Table 2).

**Figure 6:**
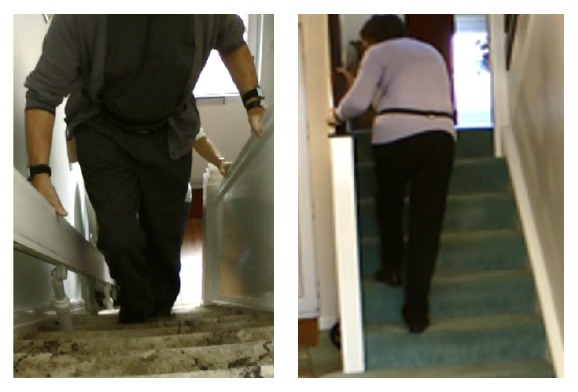
Reliance on banisters and rails. Participants utilised every available support when tackling the stairs. In the absence of a banister on both sides of the stair case, one participant kept a hand on the stairlift track and one placed both hands on the one available rail.

All participants used support (mostly furniture) to preserve their balance; particularly when turning or rising, participants paused and either repositioned their hands or feet or aborted the action. They flexed or rotated their trunks markedly to use every available support on the stairs (see Figure 6).

Participants appeared particularly unsteady during turns and on steps, if they started to walk immediately on rising, and if they did not use support when standing or sitting down. Balance was often lost backwards, but, in walking, participants tended to stumble forwards or sideways when their feet did not clear the floor or crossed, or they tripped or froze. Unsteady transfers were characterised by swaying backwards (so that the toes lifted on standing) or by actually falling backwards into the chair (either on rising, or so violently during sitting that both feet lifted off the floor: twice a participant nearly missed the chair).

Participants unsteady walking or in standing mostly took recovery steps, grabbed something, or sat down quickly to restore their balance. When participants were unsteady transferring, the chair broke the potential fall, though on five occasions (three during transfers) another person assisted/caught the participant.

### 3.4. Reflections on the Combined Data

The participant's histories, behaviour, and thoughts, alongside the researchers' observations, suggested that monitoring five activities could identify balance protection, loss, and recovery:

1. Chair transfers.
2. Walking (through open spaces and around furniture).
3. Turning (in standing and walking).
4. Stepping onto, off, or over obstacles/steps.
5. Performing tasks in standing (e.g., conversing, cooking).

### 3.5. Missing Data

We attempted to record sensor data on eighteen visits (as we reduced data collection to reduce the burden on one participant). We collected video data every time, Kinect data 17 times (94%) as a connection between sensor and lap top failed once, and wearable data 12 times (67%) after four equipment failures and two operator errors.

## 4. Discussion

Following the principles of ethnographic research, we sought to keep the situation as natural as possible before introducing sensors and continued to engage as visitors while the sensors recorded and we noted what the participants were doing. We* experienced* the reality of living with a high risk of falling, rather than simply “observed” it and support the assertion that a subjective perspective in research is valuable and increases “the knowledge yield” [21]: we gained more insight than we could have through observing, evaluating, or questioning the participants anywhere other than at home. As in previous studies [15] we had some technical issues (with equipment failure and obscured sensors) and participants disclosed some concerns about surveillance but every insight gained at this stage will inform a programme of research that is now based on experience rather than supposition.

Some people might find researchers repeatedly observing them at home difficult to accommodate to and overly intrusive but our participants allowed us to record wherever (and whatever) we wanted: none dropped out during the study. Participants may have felt that sitting the whole time we were observing them was not what we wanted to see: it is widely accepted that being part of a study can cause people to change their behaviour (“the observer effect”). Rather than a limitation, “staging a performance” can be a strength in ethnography, wherein the findings are not the raw data but the interpretation of data in context [22]. In the current study, participants may have saved activities to demonstrate while we were recording but we still found them to be sedentary (and thus surmise that they were even more sedentary when we were absent). Furthermore, our focus on fall risk meant that we were observing unintentional balance loss, rather than anything “staged.”

### 4.1. The Challenges of Using Sensors

The residential environment poses many more challenges to movement research than does the laboratory: working in a real home brought theoretical challenges into focus. The needs of residents and researchers can seem contradictory, when, for example, environmental features that assisted the residents obscured the camera's view. Some people at risk of falling rely on “furniture creeping” to negotiate safely a route around their homes, so it would be inappropriate for researchers to manipulate “obstructions” (like carefully placed chairs). However, environmental features (like doorways and gaps between furniture pieces) also* challenge* residents. We therefore suggest that when residents are largely sedentary, perhaps restricting their opportunities to fall, cameras should focus on the few challenges they still have to tackle habitually. In the current study, the need to change direction (or level) often put people at imminent risk of falling.

When exploring the potential of in-home sensors to impact health and healthcare in future, we must consider whether we need track participants 100% of the time throughout 100% of their homes. Could we answer carefully defined questions with a few appropriate sensors operating at relevant moments in high yield locations? Our findings suggest that a wearable device coupled with cameras in the sitting room and hall could meet the requirements and be acceptable to residents. For clinicians to adopt such technology, however, it would need to be more user-friendly and less distracting than the iterations we utilised: we lost data before we modified the user-interfaces on the wearables and Kinect. As a combined array however, extended human observation coupled with sensors could be an effective way of understanding how people at risk of falling negotiate or avoid high-risk locations and activities at home. We suggest clinicians working with people at high risk of falling take a detailed fall history [10] and then follow up with a period of in-home monitoring, targeting the areas of most concern on an individual basis. Even having sensors in the home for one week might yield richer data than is obtainable through self-report. In the current study, we asked participants to identify an activity that they felt carried a particularly high fall risk for them and we observed this activity repeatedly (at the end of data collection when we were familiar with the participant). In a research context, this approach is an alternative to basing sensor placement on a complete fall history.

Without witnessing an event or having video to review, clinicians (such as physiotherapists) can only glean what happened before, during, and after the event from someone's recollection. Continuously recording from multiple cameras within the home is impractical but there is the potential to keep a record of* incidents* for later scrutiny. It may be possible, for example, to record for one minute and continuously delete that recording unless there is something to report. This would revolutionise the data on which clinicians base decisions. There is growing interest in using sensors to log simple gait parameters (e.g., in people walking 20 m trials in a laboratory [23]) but the current study suggests that clinicians managing fall risk need to know more than someone's stride length and velocity. Sensors could, for example, reveal fluctuations in performance under different conditions and the availability and success of saving reactions when required.

### 4.2. The Potential of Sensors to Surpass Human Observers in Monitoring Fall Risk

People recognise an individual is at risk of falling in many ways, from how they look or move to what they say. However, an individual may* feel* that they are going to fall whilst giving no obvious indications to an observer; sensors may surpass humans in being sensitive enough to detect very subtle deviations in motor behaviour. Further research is necessary but evidence suggests that triaxial accelerometers worn on the pelvis may distinguish near falls from other gait patterns observed in healthy subjects on a treadmill in a gait laboratory [1].

Extended in-home observation has multiple advantages over the one-off “home visits” used in clinical practice. With no agenda, the observer sees how the resident uses their space, how they pace activities, and how they manage tasks when their attention is on a goal, not on the task itself. Ticking off a checklist of theoretical challenges and hazards within a single session is likely to be unrepresentative: “assessing” someone descending the steps into their kitchen is unlikely to reflect how they do it when hurrying towards a saucepan that is boiling over. Over an extended period, a human observer would be costly and intrusive; sensors would be more realistic, and monitoring by sensors* alone* would remove any need to “perform” for a* human* observer. A mixed array of sensors is likely to outdo any single type in identifying balance protection, loss, or restoration, though a single sensor has advantages in ease of application.

Without intruding in parts of the home people might prefer to keep private, sensors could monitor the risks of falling, and the associated risks of inactivity and isolation. With reasonable reservations, our participants accepted the technology. They appeared at greatest risk of falling transferring, crossing spaces without handholds, manoeuvring around obstacles, turning through doorways, and negotiating steps. These challenges are amenable to recording and most, if not all, arise along the short route between favourite chair and stair, negating the need for pervasive sensors throughout the home, with no possibility of “escape.” Having identified key activities during which experienced observers noted “instability,” our next step will be to examine the data recorded by the Kinect and wearable sensors that the participants allowed us to trial. Researchers need to establish that sensors can identify instability during simple isolated actions and then to validate the sensor-based identification of fall risk during complex free-living activities.

### 4.3. Wider Applications

Our research focusses on monitoring fall risk among people with balance and mobility disorders that restrict their function and participation in society. But our recommendation for focused (nonpervasive) monitoring applies to anyone whose health limits their activity towards a single favoured location, with everything they need close to hand. Though we studied only people with Parkinson's in the current project, the indications of fall risk that we have suggested sensors could identify and monitor are generic (with the probable exception of freezing). Although our participants were sedentary, they were still mobile: we believe that focussing sensors on a defined, frequently occupied, daytime location in the home should be as informative about how and when less sedentary (even active or impulsive) individuals move. However, unobtrusive sensors capable of identifying instability could have even wider applications. For example, alcohol intoxication may alter behaviour and fall risk. It might be possible to monitor remotely indicators of instability, if it is intrusive to monitor an individual or impossible to monitor a crowd.

## 5. Conclusions

Immersed in the reality of living with the experience, fear and risk of falling, we gained insights we would not have gained any other way. Participants enhanced their safety using banisters and grab rails but more frequently additional support in the form of strategically placed furniture. Researchers cannot remove such “obstacles” but must take their cue from them: these are where people are likely to appear unstable. Because physical obstructions, including other people, frequently obscured the cameras' views, comprehensive monitoring would necessitate multiple cameras plus at least one wearable device. However, the extent to which participants restricted their activity suggests that, by identifying an individual's high-use locations and focussing only on them, researchers and clinicians could leave the remainder of people's homes sensor-free.

We most frequently noted a high risk of falling when people transferred between sitting and standing, walked, turned, negotiated steps, and tackled tasks in standing. We noted that someone was protecting, losing, or restoring their balance largely through visual clues, so we believe that appropriately positioned sensors would also be able to detect indications of instability. Whether sensors can equal, or even surpass, human observers requires further research. People may be less likely to alter their behaviour for a sensor than for a human observer, and sensors might be able to detect changes in stability too subtle for human observers to notice in real time or from video. There is considerable scope for sensors to usefully monitor changing fall risk (rather than merely detect falls) unobtrusively. We suggest that researchers explore the value of monitoring a habitual route (such as between chair and stair, or for people living in homes without stairs, the daytime route between chair and toilet door) when evaluating fall risk over time.

## Figures and Tables

**Table 1:** Characteristics of the participants, their fall histories, and video records.

	ID-1	ID-2	ID-4	ID-5	ID-6
Age (years), gender	72, male	79, male	71, female	76, female	73, female

Parkinson's					
Years diagnosed	11	5	13	7	8
Severity (Hoehn and Yahr [20])	IV, severe	IV, severe	IV, severe	III, moderate	III, moderate

Living arrangements	With wife Needs help to leave (semidetached) house Uses mobility scooter	With wife Needs help to leave (semidetached) house Uses mobility scooter	With husband Usually only leaves (detached) house with help	With husband Usually only leaves (detached) house with help	Alone, family nearby Needs help to leave (single storey) house

Mobility	Uses stick, grab rails, and perching stool Limited stair climbing	Marked fluctuation and freezing; using riser chair; limited stair climbing (having stairlift)	Marked fluctuation Little use of aids	Little use of aids	Uses perching stool and trolley No stairs at home

Recent fall history					
Falls	>12/year	>12/year	>1/year	>1/year	0
Fractures	Yes	No	Yes	Yes	n/a
High-risk activity	Walk through kitchen-diner, negotiating step	Walk from armchair, across hall to toilet	Negotiating stairs	Negotiating stairs	Walk across open space in sitting-dining room

Video review					
Instability noted (walking/standing/transfers)	38 times in 36 min, 1.1/min (13/14/11)	86 times in 62 min, 1.4/min (66/1/19)	33 times in 19 min, 1.7/min (13/1/19)	49 times in 71 min, 0.7/min (22/12/15)	21 times in 58 min, 0.4/min (9/5/7)

min = minutes.

**Table 2:** Summary of key observations from all participants' video records, frequency observed by activity.

	Walking	Standing	Sitting to standing	Standing to sitting
Fall prevention				
Used arms			×58	×33
Held/cruised furniture	×25	×21	×2	
Held banisters/rails	×25			
Held kitchen counter	×3	×14	×1	
Held wall with free hand(s)	×13	×4		
Used stick	*1 × throughout*	×2 (leaned on)	×6	
Pause/adjust midway	×14 (turns, in space, steps)		×13	×8
Aborted attempt		×1 (to reach)	×6	
Observed instability				
“Sway” or “wobble” (e.g.)	×51 (turns, steps)	×21 (pointing, reaching)	×20 (walked straight away)	
No control/heavy (no hands)				×23/×12
Stepped/swayed backward		×6 (step(s) back)	×11 (toes off floor)	
Fell backward			×5 (into chair)	×11 (feet off floor)
Shuffled feet	×25			
Stumbled or caught foot	×13	×1		
Froze	×13			
Feet crossed	×8			
Balance recovery				
Staggered	×15	×1	×2	
Grabbed furniture	×5	×3	×2	
Grabbed wall	×2			
Grabbed banister/rail	×2			
Sat quickly	×1	*×1 with help*		*×1 with help*
*Caught by researcher *	*×1*		*×1 *	
*Quickly given stick*			*×1*	
